# Anti-Apoptotic Signature in Thymic Squamous Cell Carcinomas – Functional Relevance of Anti-Apoptotic BIRC3 Expression in the Thymic Carcinoma Cell Line 1889c

**DOI:** 10.3389/fonc.2013.00316

**Published:** 2013-12-31

**Authors:** Bei Huang, Djeda Belharazem, Li Li, Susanne Kneitz, Philipp A. Schnabel, Ralf J. Rieker, Daniel Körner, Wilfred Nix, Berthold Schalke, Hans Konrad Müller-Hermelink, German Ott, Andreas Rosenwald, Philipp Ströbel, Alexander Marx

**Affiliations:** ^1^Pathologisches Institut der Universität Würzburg, Würzburg, Germany; ^2^Pathologisches Institut und Zentrum für Medizinische Forschung (ZMF), Universitätsmedizin Mannheim der Universität Heidelberg, Mannheim, Germany; ^3^Pathologisches Institut der Universitätsmedizin Göttingen, Göttingen, Germany; ^4^Pathologisches Institut, Universität Heidelberg, Heidelberg, Germany; ^5^Abteilung Thoraxchirurgie, Thoraxklinik Rohrbach, Universität Heidelberg, Heidelberg, Germany; ^6^Neurologische Universitätsklinik Mainz, Mainz, Germany; ^7^Neurologische Universitätsklink Regensburg, Regensburg, Germany; ^8^Pathologisches Institut, Robert-Bosch-Krankenhaus, Stuttgart, Germany

**Keywords:** thymoma, thymic carcinoma, thymus, apoptosis, gene expression, myasthenia gravis, MTCH2, targeted therapy

## Abstract

The molecular pathogenesis of thymomas and thymic carcinomas (TCs) is poorly understood and results of adjuvant therapy are unsatisfactory in case of metastatic disease and tumor recurrence. For these clinical settings, novel therapeutic strategies are urgently needed. Recently, limited sequencing efforts revealed that a broad spectrum of genes that play key roles in various common cancers are rarely affected in thymomas and TCs, suggesting that other oncogenic principles might be important. This made us re-analyze historic expression data obtained in a spectrum of thymomas and thymic squamous cell carcinomas (TSCCs) with a custom-made cDNA microarray. By cluster analysis, different anti-apoptotic signatures were detected in type B3 thymoma and TSCC, including overexpression of BIRC3 in TSCCs. This was confirmed by qRT-PCR in the original and an independent validation set of tumors. In contrast to several other cancer cell lines, the BIRC3-positive TSCC cell line, 1889c showed spontaneous apoptosis after BIRC3 knock-down. Targeting apoptosis genes is worth testing as therapeutic principle in TSCC.

## Introduction

Thymomas comprise a spectrum of unique thymic epithelial tumors that generally show intratumoral thymopoiesis. They are subdivided into WHO type A, AB, B1, B2, and B3 thymomas (1, 2), but this classification has been challenged by some authors (3, 4). Thymic carcinoma also show a spectrum subtypes that resemble analogously called extrathymic carcinomas (TCs) (2, 5). However, there is strong evidence (6–13) that thymic squamous cell carcinomas (TSCCs) and other squamous cell carcinomas are different entities. Nevertheless, treatments tailored to the unique biology of thymomas and TCs are missing (14–16) since there are almost no therapeutic targets (9–13, 17, 18), with few exceptions (e.g., Kit mutations) (12, 13). Even whole genome sequencing of a stage IVa B3 thymoma (19) and sequencing of 46 cancer genes in a TSCC (20) discovered no druggable mutations, suggesting that pathways other than in more common cancers might be operative (19). Considering this information and reports about the expression of apoptosis-related proteins in thymic tumors (21) we analyzed our unpublished gene expression data of thymuses and thymic tumors that were obtained with a custom-made cDNA microarray (representing 4606 genes). We found differential expression of anti-apoptotic genes in B3 thymomas and TSCC and could induce apoptosis by BIRC3 blockade in the thymic carcinoma cell line, 1889c (22).

## Patient Characteristics and Methods

### Patients and tissues

Characteristics of the *historic* tumors (resected before 2004) stemmed from our data base (23) (Table 1). For the validation set of *recent* tumors obtained after 2006 see Table 2. Thymoma classification and staging followed the WHO and modified Masaoka system, respectively (2). All carcinomas were TSCCs without prior chemotherapy. “Combined thymomas” with >10% separable components were excluded. Thymocytes from a normal thymus were purified by Ficoll density gradient centrifugation. Ethical approval was obtained.

**Table 1:** **Characteristics of patients and tissues: WHO type A, AB, B2, B3 thymomas; TSCC, thymic squamous cell carcinoma; NT, normal thymus; thymitis, non-neoplastic thymus with lymphofollicular hyperplasia (LFH) from early-onset Myasthenia Gravis (MG) patients; thymocytes, purified from NT; MG+ (%), %-age of patients with MG**.

Diagnosis	*N*	Age range (years)	Sex (m:f)	Masaoka stage (I–IV)	MG+ (%)	Follow-up
Thymoma
Type A	7	58–77	5:2	I (*n* = 3)	3 (43%)	All alive (2/2)
				II (*n* = 4)	
Type AB	16	33–79	5:11	I (*n* = 6)	10 (62%)	All alive (9/9)
				II (*n* = 10)	
Type B2	15	38–78	8:7	I (*n* = 1)	9 (60%)	3 of 7 DOD
				II (*n* = 7)	
				III (*n* = 6)	
				IV (*n* = 1)	
Type B3	13	33–73	7:6	I (*n* = 0)	10 (76%)	3 of 11 DOD
				II (*n* = 5)	
				III (*n* = 5)	
				IV (*n* = 3)	
TSCC	8	44–71	5:3	I (*n* = 0)	0	4 of 8 DOD
				II (*n* = 2)	
				III (*n* = 2)	
				IV (*n* = 4)	
NT	7	1–38		–	0	–
Thymitis	7	32–37		–	7	–
Thymocytes	1	1		–	0	–

*DOD, death of disease; n.k., not known*.

**Table 2:** **Characteristics of 36 recent WHO type A and B3 thymomas and TSCCs that served as validation set for cases from Table 1**.

Diagnosis	*N*	Age range (years)	Sex (m:f)	Masaoka stage (I–IV)	MG+ (%)	Follow-up
Thymoma
Type A	7	71–87	4:3	I (*n* = 2)	1 (14, 3%)	unknown
				II (*n* = 5)		
Type B3	18	35–80	8:12	I (*n* = 2)	6 (33%)	Partially known
				II (*n* = 6)		2: dead
				III (*n* = 4)		1: alive
				IV (*n* = 6)		
TSCC	11	40–74	9:2	I (*n* = 1)	0	Partially known
				II (*n* = 6)		1: dead
				III (*n* = 2)		1: alive
				IV (2)		

### Gene expression profiling

Gene expression profiling was achieved with a custom cDNA microarray representing 4606 genes with known relationship to cancers. To study reproducibility, 11 of 74 biopsies were studied in duplicate. Differential gene expression was analyzed by ANOVA (JMP Genomics, version 4; SAS, Cary, NC, USA).

### RT-PCR

Array-based gene expression in the historic tumors was re-evaluated by qRT-PCR (TaqMan; FAST SYBR Green; Applied Biosystems) (Table S1 in Supplementary Material). Relative quantification was calculated using the ΔΔCt method with GAPDH as standard (Figure S2 in Supplementary Material). For primers and condition see Table S1 in Supplementary Material. Confirmed genes were checked by qRT-PCR in the validation set of 36 tumors.

### Pathway analysis

Pathways were studied using Gene Set Enrichment Analysis (GSEA) (24). Statistical significance (nominal *p*-value, NP) of enrichment scores (ES) was estimated using 1000 rounds of permutations per pathway. To adjust for multiple hypothesis testing, ES were normalized (“normalized enrichment scores,” NES) taking pathway size into account. The proportion of false positives was controlled by calculating the false discovery rate (FDR) for each NES.

### Cell lines and transfection with si-BIRC3

BIRC3^+^ human cell lines studied here: 1889c (thymic carcinoma) (22); HaCat (keratinocytes); PC3 (prostate carcinoma); TE167 (rhabdomyosarcoma); A549 (lung carcinoma); MCF7 (breast carcinoma); HeLa (cervical adenocarcinoma). Culture conditions: RPMI (1889c) or DMEM (all others); 10% fetal bovine serum, 2 mM l-Glutamine and penicillin/streptomycin; 37°C; 5% CO_2_. About 10^5^ cells transfected with 2 μM BIRC3 siRNA FlexTube: si00022827 (Qiagen, Hilden, Germany) or scramble siRNA using High-Perfect transfection reagent (Qiagen; fast forward transfection protocol) were harvested after 48 h.

### Western blot, immunocytochemistry, and apoptosis detection

Cell lines and snap frozen tissues were used for Western blot (25). Methanol-fixed si-BIRC3 transfected cells and scramble siRNA transfected controls were stained with mouse anti-human cleaved caspase 3 (1:100), pH 6, Abcam for 1 h (25).

About 10^5^ transfected cells including floating cells were harvested, stained at 20°C for 10 min with annexin V-FITC and Propidium Iodide PI (BD Biosciences), and evaluated for apoptosis by flow cytometry.

## Results

### Distinct gene expression profiles correlate with WHO-defined thymoma subtypes

**Figure 1:**
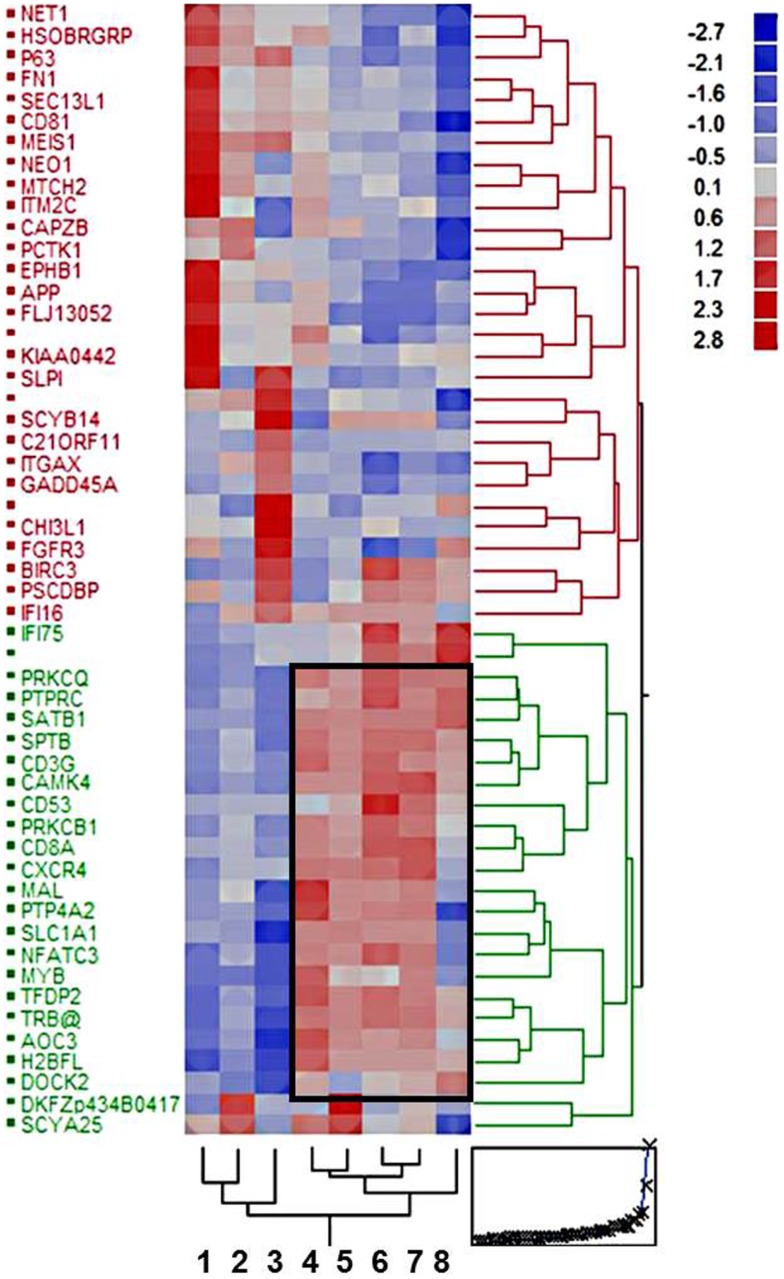
**Hierarchical clustering of eight tissue cohorts: type A, AB, B2, and B3 thymomas; thymic squamous cell carcinomas (TSCCs); non-neoplastic thymuses (normal and MG-associated thymitis); and Ficoll gradient-purified thymocytes based on 53 differentially expressed genes (*p* < 10^−10^)**. The frame delineates 19 genes with coordinate up-regulation in the lymphocyte-rich cohorts, most of which (*n* = 13) play a role in lymphocyte biology (for details see text).

Gene expression profiling of eight cohorts, i.e., type A, AB, B2, B3 thymomas, TSCC, normal thymus, thymuses with thymitis, and thymocytes revealed 53 differentially expressed gens (*p* < 10^−10^). Based on these genes the cohorts were grouped into two major clusters: an epithelial-rich cluster with type A and B3 thymomas, and TSCCs, and a lymphocyte-rich cluster with AB, B2 thymomas, thymuses, and thymocytes (Figure 1). Clustering was closer between type A and B3 thymomas than between TSCC and B3 thymomas (Figure 1), despite the similar malignant potential of B3 thymomas and TSCC (22, 26, 27). Thymuses clustered closer to B2 than to AB thymomas, which may echo the more physiological thympoiesis in B2 thymomas (28, 29). GSEA (24)^1^ of genes over-expressed throughout the lymphocyte-rich cohorts showed that 13 out of 19 annotated genes (Frame in Figure 1) play a role in T cell biology (*p* < 10^−12^).

### Functional pathways enriched in epithelial-rich thymic tumors

To reduce the impact of confounding thymocytes, we focused GSEA on type A and B3 thymomas and TSCCs. Using NES of >1.6 and *p* < 0.05 between at least two comparisons (24), we retrieved 11 functional pathways (Table 3). All three tumor cohorts were differentially enriched by all pair-wise comparisons for pathways comprising DNA damage response genes. Since enrichment increased from TSCCs to type A thymomas, this may reflect the highest genetic instability in TSCCs and lowest in type A thymomas (9, 10). Type A thymomas were enriched for three pathways: among the “cell migration genes,” over-expressed KAl1 has anti-metastatic function (30); IL8RB reinforces p53-dependent senescence (31); and SAA1 expression that has not been noted in thymomas before, can have pro- or anti-apoptotic functions in context-dependent manner (32–36). Among the “transcriptional corepressor genes” the tumor suppressor gene ID4 was over-expressed in type A thymomas; its reduction – as in type B3 thymomas and TSCCs – is typical of various cancers (37). These findings seemingly reflect the lower malignant potential of type A thymomas (23, 27).

**Table 3:** **Significantly enriched functional pathways in type A and B3 thymomas and TSCC using normalized enrichment scores (NES) of >1.6 and *p* < 0.05 between at least two comparisons; NP, normalized *p*-values (24). The “exemplary genes” are the genes with the strongest (>1.3-fold) significant (*p* < 0.05) differential expression in the indicated Gene Ontology pathway. Genes given in bold are discussed in the text**.

Typical for	Pathway (gene ontology)	Exemplary genes	B3_A		TSCC_A		TSCC_B3	
			NES	NP	NES	NP	NES	NP
All	DNA damage response signal transduction by P53 class mediator	*IFI16*	1.74	0.01	1.87	0.00	1.71	0.01
A	Cell migration	**IL8RB, KAL1, SAA1**	−1.70	0.01	−1.84	0.00	−0.90	0.61
	Transcriptional corepressor activity	**ID4**	−1.70	0.01	−1.76	0.00	−1.12	0.31
	Cytoplasm organization and biogenesis	*ITGA6*	−1.77	0.00	−1.66	0.00	−0.92	0.57
B3	Structure molecule activity	*RPS18*	−1.98	0.00	0.90	0.67	2.13	0.00
	Structural constitute of ribosome	**RPS6, RPS18**	−2.28	0.00	0.78	0.81	2.45	0.00
	Translation		−1.87	0.00	0.60	0.98	1.79	0.00
	ADP binding		−1.71	0.01	0.82	0.70	1.90	0.00
	RNA binding	*RPS6*	−1.80	0.00	−0.64	0.97	1.74	0.01
C	Caspase activation	**PMAIP1**	1.02	0.45	1.67	0.03	1.90	0.00
	Antigen binding	*IGHG3*	0.74	0.78	1.66	0.01	1.66	0.02

In B3 thymomas, most of the enriched pathways concerned translation, including down-regulation of the ribosomal protein genes RPS6 and RPS18, which is rare in other cancers (38). Since RPS6 is an effector of the AKT/mTOR pathway, we studied expression of its negative regulators PTEN and PIK3R1 but found them up-regulated only slightly (*p* < 10^−3^) in B3 thymomas. Vice versa, up-regulation of RPS6 and down-regulation of PTEN and PIK3R1 in TSCCs is typical of aggressive cancers (39, 40).

For the role of caspase activity/apoptosis-related genes in TSCCs see next paragraph.

### Apoptosis-related signature in TSCC

**Figure 2:**
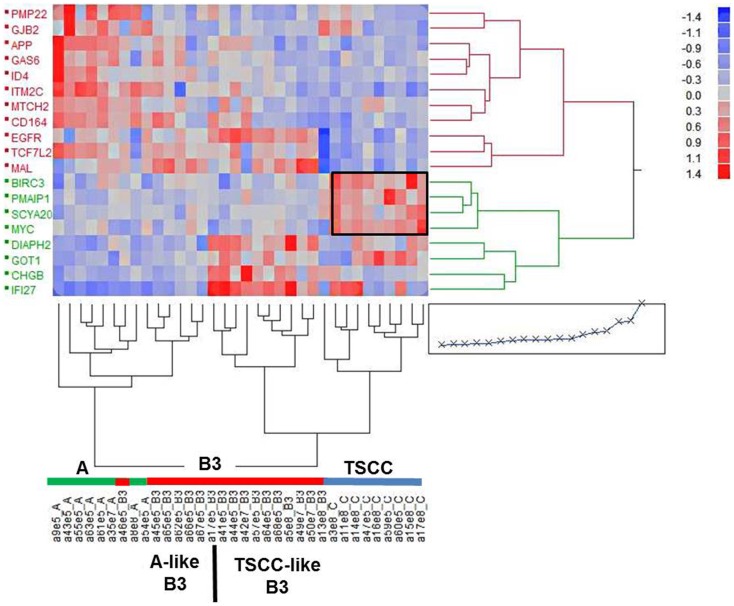
**Cluster analysis of 19 expressed genes in individual epithelial-predominant type A and B3 thymomas and TSCC based on a fold-change >1.5, *p* < 10^−4^ and low variance (var < 0.07) in at least one of the cohorts of tumors**. One of the two main clusters (left) harbors all of the type A thymomas and six of the (“type A-like”) B3 thymomas. The other cluster harbors all the TSCC and 11 of the (TSCC-like) B3 thymomas. The frame highlights a cluster of apoptosis-related genes (BIRC3, SCYA20, PMAIP1, MYC, MTCH2).

**Figure 3:**
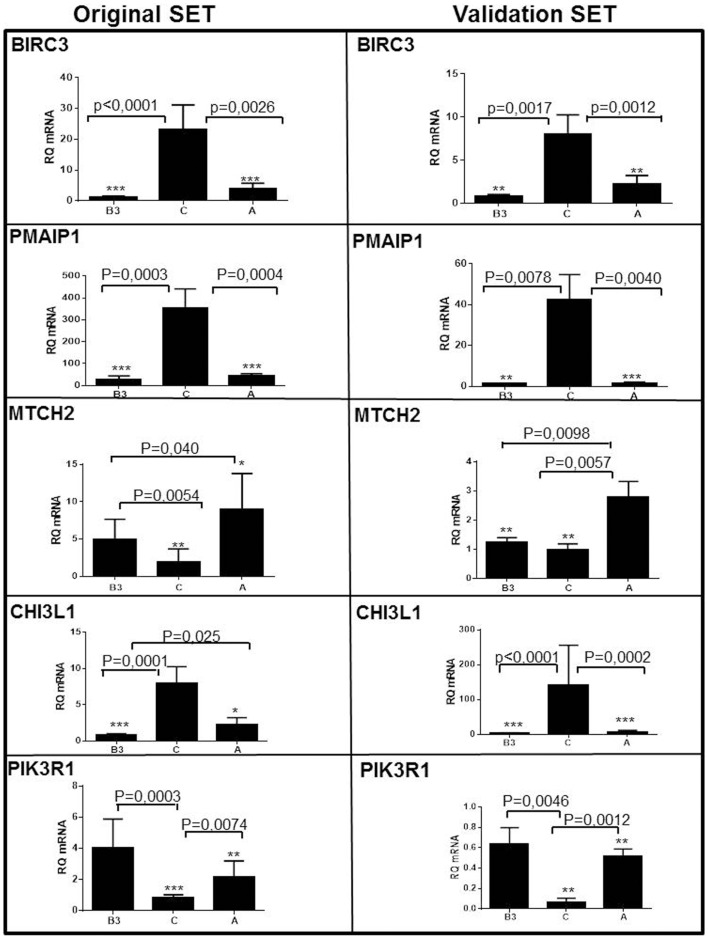
**Validation of five differentially expressed genes in type A and type B3 thymomas and thymic squamous cell carcinomas (TSCC, C) in the original set of tumors (left column; see Table 1) and the validation set (right column; see Table 2)**. *BIRC3*, *PMAIP1/NOXA*, and *Chitinase3-like1* genes are over-expressed in TSCC. The pro-apoptotic *MTCH2* is expressed in type A thymomas (and largely missing in TSCC). PIK3R1 is most significantly expressed in type B3 thymomas.

Nineteen genes were differentially expressed between type A and B3 thymomas and TSCC (>1.5-fold and *p* < 0.0001) and with low variation (var < 0.07) in at least one tumor subtype (Figure 2, Table S1 in Supplementary Material). By hierarchical clustering, type A thymomas and TSCCs formed distinct groups, while 6 out of 17 B3 thymomas were clustered with the type A thymomas and 11 with the TSCCs (Figure 2). The B3 thymoma subsets were not different with respect to age, sex, and tumor stage (not shown) but 9 of 10 myasthenia-associated B3 thymomas belonged to the “TSCC-like” subset. Figure 2 also depicts apoptosis-related genes that are co-up-regulated in TSCCs, two with anti-apoptotic (BIRC3; SCYA20) and two with pro-apoptotic function (PMAIP1; MYC). Importantly, the pro-apoptotic MTCH2 gene (41, 42) was down-regulated in TSCCs as also confirmed by qRT-PCR (Figure 3, left column; Table S1 in Supplementary Material) and validated in more recently obtained tumors (Figure 3, right column).

### Knock-down of BIRC3 induces apoptosis in thymic carcinoma cell line 1889c

**Figure 4:**
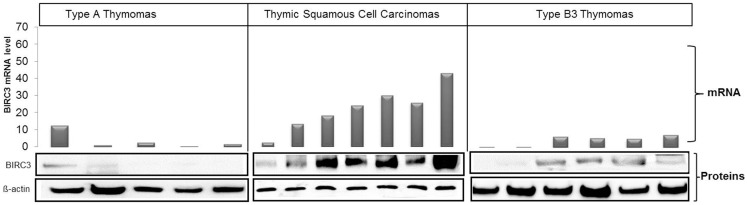
**Correlation between BIRC3 mRNA and protein levels in type A (five cases) and type B3 (six cases) thymomas and TSCC (seven cases)**. Levels of mRNA and protein were higher in TSCCs than in thymomas. mRNA was quantified using real time PCR with GPDH as reference, β-actin was used as loading control in western blots.

**Figure 5:**
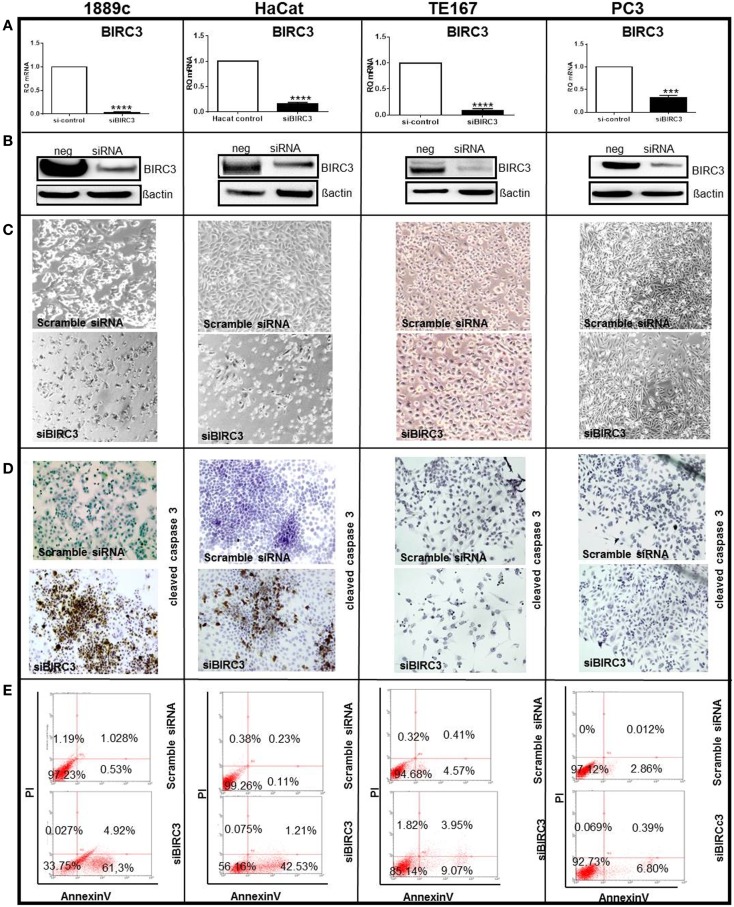
Several cell lines (1889c, HaCat, TE167, and PC3) were transfected using si-BIRC3 for 48h, which significantly repressed expression of (A) Birc3 mRNA ****p < 0.0001 in 1889c, HaCat and TE167 cells and ***p = 0.0002 in PC3) and (B) Birc3 protein. After transfection. Changes of the cytology of transfected cells is shown in **(C)**. Cells were stained with an antibody to cleaved caspase 3 for apoptosis detection (immunoperoxidase ×20) **(D)**. FACS analysis to quantify apoptosis using Annexin V-FITCS/PI **(E)**. All experiments were done in triplicate and each measurement in duplicate. Similar low levels of apoptosis following si-BIRC3 transfection were observed in A549 (lung carcinoma); MCF7 (breast carcinoma); and HeLa (cervical adenocarcinoma) cells.

Among differentially expressed genes, we selected BIRC3 for functional analysis, because (i) it is a member of the inhibitors of apoptosis (IAPs) gene family (43) that serves key oncogenic roles in many cancers (44, 45); (ii) BIRC3 protein was over-expressed in parallel with mRNA in most TSCC biopsies compared to thymomas (Figure 4); and, (iii) the thymic carcinoma cell line, 1889c (22) with strong BIRC3 expression was available (Figure S2 in Supplementary Material). siRNA-mediated BIRC3 knock-down (Figures 5A,B) induced spontaneous apoptosis in 50% of 1889c cells within 48 h as shown by cytology, expression of activated caspase 3 and annexin 5 (Figures 5C–E). 1889c cells and HaCat keratinocytes were more sensitive toward BIRC3 knock-down than all other cancer cell lines tested (Figures 5A–E; Figure S3 in Supplementary Material), despite similar BIRC3 mRNA levels (Figure S2 in Supplementary Material).

## Discussion

The major finding here is the so far unreported (13, 19, 46) observation of different expression profiles of apoptosis-related genes in TSCCs and B3 thymomas in two independent sets of thymic epithelial tumors. Significantly, anti-apoptotic BIRC3 – a member of the “IAPs” gene family that mainly blocks the *extrinsic* apoptosis pathway – was over-expressed at the mRNA and protein level in most TSCCs compared to type A and B3 thymomas; and BIRC3-positive 1889c thymic carcinoma cells showed stronger spontaneous apoptosis on BIRC3 knock-down *in vitro* than all other cell lines tested. In addition, other than the thymomas, the TSCCs showed lower mRNA expression of the *MTCH2* gene that induces apoptosis by cooperation with tBID to facilitate BAX-mediated mitochondrial cytochrome c release (47). These findings suggest that TSCCs suffer from attenuation of both the extrinsic and intrinsic apoptosis pathway. This is complemented in TSCCs by down-regulation of *PIK3R1* (Figure 3) that normally interferes with various pro-survival cascades, including Akt signaling (40, 41). By contrast, the anti-apoptotic make-up of B3 thymomas in the current study was due to the down-regulation of the pro-apoptotic *PMAIP1/NOXA* gene (Figure 3) that plays a role in the mitochondrial apoptosis pathway and drug resistance (48).

A caveat here is the limited coverage of the transcriptome by our applied microarray. While key apoptosis-related gens like *BCL2*, *MCL1*, *BIRC2*, *BIRC5*, *BCL2L1* were represented but did not show differential expression between TSCC and B3 thymomas (not shown), other important genes (e.g., *BIRC4/XIAP*, *BIRC6-8*, *BAX*, *BBC3/PUMA*, and *BIM*) were missing and warrant further analysis. Despite these limitations, our findings could have a translational perspective, since advanced tumor stage, limited resectability, and common relapses (13–15, 23, 26, 49–51) are frequent indications in TSCCs for (neo-)adjuvant therapies that may be critically attenuated by apoptosis resistance of the target tumor cells (52). Indeed, therapeutic options are already available (e.g., #NCT00977067; #NCT01078649)^2^ or up-coming (53), including drugs against BIRC3 (54) and strategies to induce *PMAIP1* (48). RT-PCR-based expression profiles of anti-apoptotic genes are worth testing as “biomarkers” in clinical trials aiming to break treatment resistance in thymic cancers.

## Conflict of Interest Statement

The authors declare that the research was conducted in the absence of any commercial or financial relationships that could be construed as a potential conflict of interest.

## Supplementary Material

The Supplementary Material for this article can be found online at http://www.frontiersin.org/Journal/10.3389/fonc.2013.00316/abstract

Table S1 **Characteristics of the 19 genes with significantly different expression in epithelial-rich thymic epithelial tumors (Figure 2): type A and B3 thymomas and TSCCs**. Functions are: AD, adhesion; AP, apoptosis; Diff, differentiation; IR, immune response; M, migration; P, proliferation; TD, T cell development; IF, inflammation; TR, tissue remodeling. The differential expression of all genes in the original set of tumors was confirmed by qRT-PCR (see also Figure 3).

Table S2 **Primer sequences and annealing temperatures used to confirm and validate by qRT-PCR the expression of those genes that were found to be differentially expressed on microarray analysis and are depicted in Figure 3 and Figure S1 in Supplementary Material**.

Figure S1 **Confirmation and validation of cKIT gene expression using qRT-PCR in TSCC**.

Figure S2 **Semi-quantitative determination of BIRC3 mRNA levels in several cell lines using standard PCR with 10 ng cDNA as template for each sample**. BIRC3 expression was detectable in all cell lines except the rhabdomyosarcoma cell lines RH30 and CRL2061. GAPDH was used as control.

Figure S3 **Evaluation of annexin V-FITC/PI labeled cells using FACS 48 h after BIRC3 knock-down**. The transfected 1889c thymic carcinoma cells showed the highest level of apoptosis (50%) compared to HaCat (40%), TE167 (10%, **p = 0.0097), A459 (20%, *p = 0.0432), PC3 (<10%, **p = 0.0082), MCF7 (<20%, *p = 0.0154), and HeLa (<10%, **p = 0.0079). The results represent the mean of three independent experiments, each with duplicate measurements.

## Footnotes

^1^http://www.broadinstitute.org/gsea/index.jsp

^2^www.ClinicalTrials.gov
