# Supplementary material for: Anti-Apoptotic Signature in Thymic Squamous Cell Carcinomas – Functional Relevance of Anti-Apoptotic BIRC3 Expression in the Thymic Carcinoma Cell Line 1889c
Source: Front Oncol. 2013 Dec 31;3:316. doi: 10.3389/fonc.2013.00316 (PMC3876280; doi:10.3389/fonc.2013.00316)
Supplement: Table S1 — Characteristics of the 19 genes with significantly different expression in epithelial-rich thymic epithelial tumors (Figure 2): type A and B3 thymomas and TSCCs. Functions are: AD, adhesion; AP, apoptosis; Diff, differentiation; IR, immune response; M, migration; P, proliferation; TD, T cell development; IF, inflammation; TR, tissue remodeling. The differential expression of all genes in the original set of tumors was confirmed by qRT-PCR (see also Figure 3). [file 71458_Marx_DataSheet1.DOC]

Table S1. Characteristics of the 19 genes with significantly different expression in epithelial-rich thymic epithelial tumors (Fig. 2): type A and B3 thymomas and TSCCs. Functions are: AD, adhesion; AP, apoptosis; Diff, differentiation; IR, immune response; M, migration; P, proliferation; TD, T cell development; IF, inflammation, TR, tissue remodeling. The differential expression of all genes in the original set of tumors was confirmed by qRT-PCR (see also Fig. 3).

| **Gene**  Locus | **Name(s)** | **P** | **Function** | **Main Expression**  **(WHO Type)** |
| --- | --- | --- | --- | --- |
| **PMP22**  17p11 | Peripheral Myelin Protein 22 | **10^-4.1^** | **AD, M** | **A > B3** |
| **GJB2**  13q11-q12 | Gap Junction Protein-Beta2, Connexin 26 | **10^-5.9^** | **P** | **A > B3** |
| **APP**  21q21 | Amyloid Beta A4 Precursor Protein | **10^-7.4^** | **P, Diff** | **A > B3** |
| **GAS6**  13q34 | Growth-arrest Specific 6, AXL-L | **10^-6.8^** | **P, AP** | **A > TSCC** |
| **ID4**  6p22-p21.3 | Inhibitor of DNA Binding 4 | **10^-4.9^** | **P, Diff** | **A > B3** |
| **ITM2C**  2q37 | Integral Membrane Protein 2c, BRI3 | **10^-12.6^** | **AP** | **A > B3** |
| **MTCH2**  11q12.1 | Mitochondrial Carrier Homologue 2 (pro-apoptotic function) | **10^-10^** | **AD, AP** | **A > B3** |
| **CD164** 6p21 | Endolyn, Sialomucin CD164 | **10^-4.2^** | **P, AD, M, Diff** | **A > B3** |
| **EGFR** 7p12.3-p12.1 | Epidermal Growth Factor Receptor ERBB1, HER1 | **10^-5.6^** | **P** | **B3, A > TSCC** |
| **TCF7L2**  10q25.3 | Transcription Factor 7-like 2, TCF4 (context dependent proto-oncogene or tumor suppressor) | **10^-8.9^** | **P, M, Diff** | **B3, A > TSCC** |
| **MAL**  2cn-q13 | Myelin and Lymphocyte Protein | **10^-9.7^** | **P, M, AP** | **B3, A > TSCC** |
| **BIRC3** 11q22-q23 | Baculoviral IAP Repeat-Contain-ing Protein 3; cIAP2 (inhibitor of apoptosis 2) | **10^-8.5^** | **AP** | **TSCC > A, B3** |
| **PMAIP1** 18q21.32 | PMA Induced Protein (Noxa)  Activation of caspases | **10^-5.9^** | **AP** | **TSCC > A, B3** |
| **SCYA20**  2q32-q37 | Chemokine, CC Motif, Ligand 20, CCL20 | **10^-5.6^** | **P, M, AP** | **TSCC > A, B3** |
| **MYC**  8q24.12-q13 | V-MYC Avian Myelomonocyto-matosis Viral Oncogene Homolog | **10^-4.1^** | **P, AP** | **TSCC > A, B3** |
| **DIAPH2** Xq22 | Diaphanous, Drosophila, Homolog of; DIA2 | **10^-6.0^** | **AD, M,** | **B3 > TSCC** |
| **GOT1** 10q24.1-25.1 | Glutamate Oxalacetate Transami-nase, Aspartate Amino Transferase | **10^-6.3^** | **P** | **TSCC,B3 > A** |
| **CHGB** 20pter-p12 | Chromogranin B | **10^-5.9^** | **Diff** | **B3 > TSCC** |
| **IFI27** 14q32 | Interferon Alpha Inducible Protein 27 | **10^-8.4^** | **AP, IR** | **B3 > TSCC** |
