# Supplementary material for: Anti-Apoptotic Signature in Thymic Squamous Cell Carcinomas – Functional Relevance of Anti-Apoptotic BIRC3 Expression in the Thymic Carcinoma Cell Line 1889c
Source: Front Oncol. 2013 Dec 31;3:316. doi: 10.3389/fonc.2013.00316 (PMC3876280; doi:10.3389/fonc.2013.00316)
Supplement: Table S2 — Primer sequences and annealing temperatures used to confirm and validate by qRT-PCR the expression of those genes that were found to be differentially expressed on microarray analysis and are depicted in Figure 3 and Figure S1 in Supplementary Material. [file 71458_Marx_DataSheet2.DOC]

**Table S2**: Primer sequences and annealing temperatures used to confirm and validate by qRT-PCR the expression of those genes that were found to be differentially expressed on microarray analysis and are depicted in Fig.3 and Fig. S1.

| **Primer** | **Primer sequences** | **Annealing temp** |
| --- | --- | --- |
| BIRC3 | F: 5´-CTT TGC CTG TGG TGG AAA AT-3´  R: 5´-ACT TGC AAG CTG CTC AGG AT-3´ | 60°C |
| PMAIP1/NOXA | F: 5´-AAG AAG GCG CGC AAG AAC-3´  R: 5´-TCC TGC GCA GAA GAG TTT GGA-3’ | 60°C |
| CHI3L1 | F:  5´-TCA AGA ACA GGA ACC CCA AC-3´  R:  5´-AAA TTC GGC CTT CAT TTC CT-3´ | 60°C |
| PIK3R | F: 5´-CTG CCT CCT AAA CCA CCA AA-3´  R: 5´-TAC CAA AAA GGT CCC GTC TG-3´ | 60°C |
| MTCH2 | F: 5´-CAC ATT GCC AGT ATC GAT GG-3´  R: 5´-TGT GAT GAG GGT AGC AGC AG-3´ | 60°C |
| GAPDH | F: 5' TCGACAGTCAGCCGCATCT 3'  R: ' CCGTTGACTCCGACCTTCA 3' | 60°C |
| cKIT | F: 5'-ATGTTGTCCCAACCAAGG-3'  R: 5'-CTTCTAAGTCTAGGGCCAACTC-3' | 60°C |
