# Supplementary figures and images for: Anti-Apoptotic Signature in Thymic Squamous Cell Carcinomas – Functional Relevance of Anti-Apoptotic BIRC3 Expression in the Thymic Carcinoma Cell Line 1889c
Source: Front Oncol. 2013 Dec 31;3:316. doi: 10.3389/fonc.2013.00316 (PMC3876280; doi:10.3389/fonc.2013.00316)

## Original SET

KIT

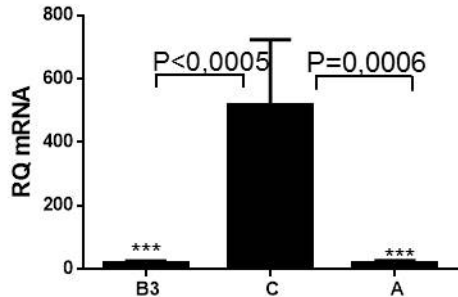

## Validation SET

KIT

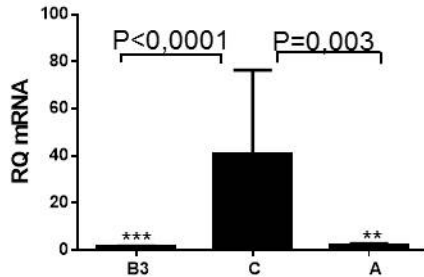

Supplement: Figure S1 — Confirmation and validation of cKIT gene expression using qRT-PCR in TSCC. [file 71458_Marx_Presentation1.PDF]

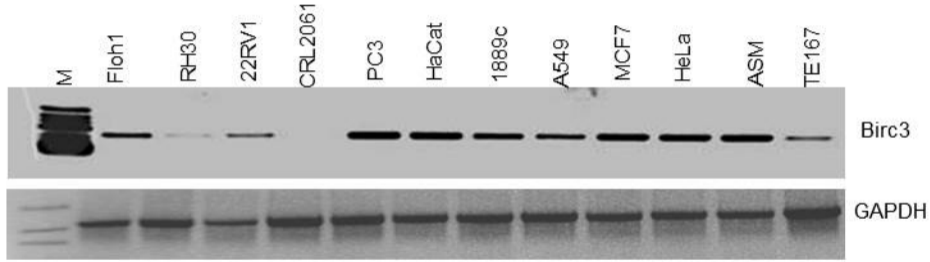

Supplement: Figure S2 — Semi-quantitative determination of BIRC3 mRNA levels in several cell lines using standard PCR with 10 ng cDNA as template for each sample. BIRC3 expression was detectable in all cell lines except the rhabdomyosarcoma cell lines RH30 and CRL2061. GAPDH was used as control. [file 71458_Marx_Presentation2.PDF]

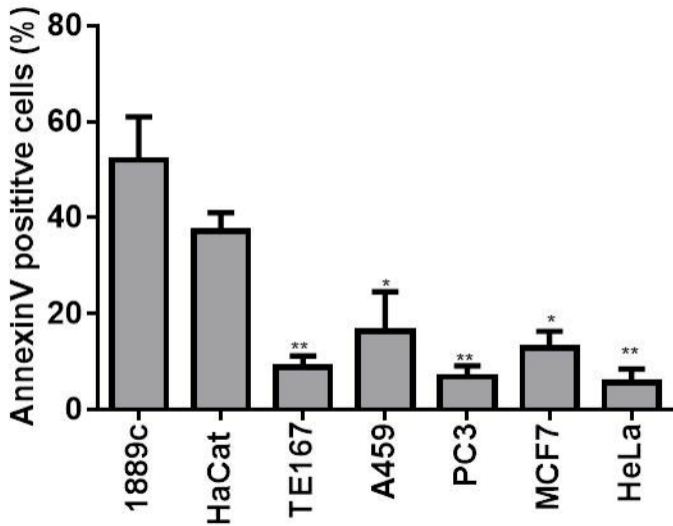

Supplement: Figure S3 — Evaluation of annexin V-FITC/PI labeled cells using FACS 48 h after BIRC3 knock-down. The transfected 1889c thymic carcinoma cells showed the highest level of apoptosis (50%) compared to HaCat (40%), TE167 (10%, **p = 0.0097), A459 (20%, *p = 0.0432), PC3 (<10%, **p = 0.0082), MCF7 (<20%, *p = 0.0154), and HeLa (<10%, **p = 0.0079). The results represent the mean of three independent experiments, each with duplicate measurements. [file 71458_Marx_Presentation3.PDF]
